# Understanding household health through data linkage.

**DOI:** 10.23889/ijpds.v7i3.1786

**Published:** 2022-08-25

**Authors:** Mai Stafford, Sarah Deeny, Kathryn Marszalek, Jenny Shand

**Affiliations:** 1 The Health Foundation; 2 UCL Partners

## Objectives

Household factors can affect the level and type of health and social care a person with long-term conditions needs. Information on household composition and health needs within households is not easily available in routine health records but linked data from health providers and local government can address this gap.

## Approach

Individual-level linked data from local government services, health providers and health commissioners in Barking and Dagenham was used. This provided sociodemographic, health and household information alongside activity data for five care settings (primary care, hospital, community, inpatient and outpatient mental health services, and social care). We identified 9222 residents aged 50+ living in two-person households between April 2016 and March 2018. Their long-term conditions were counted using primary care data. Annualised use and cost of care services were compared for households with one versus both residents having two or more long-term conditions (known as “multimorbidity”).

## Results

Over 45% of multimorbid people in two-person households were co-resident with another multimorbid person. This percentage was higher in the most deprived areas. In households where both residents were multimorbid, each resident had higher use of some types of care. After adjustment for age, gender and area deprivation, those co-resident with another multimorbid person were 1.14 (95% CI 1.00, 1.30) times as likely to have any community care activity and 1.24 (95% CI 0.99,1.54) times as likely to have any mental health care activity compared to those co-resident with a healthy person. They had more primary care visits (8.5 (95% CI 8.2,8.8) vs 7.9 (95% CI 7.7,8.2)) and higher primary care costs. Outpatient care and elective admissions did not differ between these groups.

## Conclusion

Care use for people with multimorbidity depends on the health of others in their household. Use of household health data could inform local planning for health and other community services. Its use for delivering individual or household level care could be explored, if acceptable to patients. Future research could examine larger households.

